# Key Health Information Technologies and Related Issues for Iran: A Qualitative Study

**DOI:** 10.2174/1874431101812010001

**Published:** 2018-04-30

**Authors:** Morteza Hemmat, Haleh Ayatollahi, Mohammadreza Maleki, Fatemeh Saghafi

**Affiliations:** 1 Social Determinants of Health Research Center, Saveh University of Medical Sciences, Saveh, Iran; 2 School of Health Management and Information Sciences, Iran University of Medical Sciences, Tehran, Iran; 3 School of Management, University of Tehran, Tehran, Iran

**Keywords:** Health information technology, Key technology issues, Foresight, Qualitative study, Decision making, Interoperability standards

## Abstract

**Background and Objective:**

Planning for the future of Health Information Technology (HIT) requires applying a systematic approach when conducting foresight studies. The aim of this study was to identify key health information technologies and related issues for Iran until 2025.

**Methods:**

This was a qualitative study and the participants included experts and policy makers in the field of health information technology. In-depth semi-structured interviews were conducted and data were analyzed by using framework analysis and MAXQDA software.

**Results:**

The findings revealed that the development of national health information network, electronic health records, patient health records, a cloud-based service center, interoperability standards, patient monitoring technologies, telehealth, mhealth, clinical decision support systems, health information technology and mhealth infrastructure were found to be the key technologies for the future. These technologies could influence the economic, organizational and individual levels. To achieve them, the economic and organizational obstacles need to be overcome.

**Conclusion:**

In this study, a number of key technologies and related issues were identified. This approach can help to focus on the most important technologies in the future and to priorities these technologies for better resource allocation and policy making.

## 1 INTRODUCTION

Health Information Technology (HIT) includes a variety of information and communication technologies which are used to collect, transmit, display, or store patient data [1]. This concept covers a wide range of products, technologies, and services, such as remote and mobile health (mhealth) technology, cloud-based services, medical devices, telemonitoring tools, and sensor technologies [2].

The potential benefits of Health Information Technology (HIT) include improving the quality and efficiency of health care, reducing healthcare costs [3], improving accessibility of information for patients, clinicians, health care providers, insurance organizations and other governmental departments, decreasing medical errors, and empowering patients and clinicians [4-6]. Apart from the potential benefits, the risk factors and the potential negative impacts need to be considered, too [7]. Overall, it seems to be difficult to plan for the future health information technology. In addition, HIT is developing fast [8] and policy-makers should consider the events that may happen in the years ahead [9]. Therefore, the use of systematic future research has been recommended for better planning and successful utilization of these technologies [10].

A systematic study of the future is recognized by various names, such as futures study, foresight, futurology, prognostic study, and prospective study. The aim of this type of research is to identify preferred, possible, and probable future [11, 12]. Foresight is “a systematic process with a long-term vision in scientific, technological, economic, and social areas which aims to determine strategic research areas and provide a basis for the emergence of new technologies with more benefits for community and economy” [13].

Technology foresight methods are used to determine the medium- and long-term outlooks of technology development [14, 15] and identify the key or critical technology issues of a country or an industry that should be developed to shape the desired future [16]. It is also considered as one of the most important elements of a technology development process. This process provides outputs to set technology strategies and infrastructure. In addition, technology foresight supports public and private companies in terms of innovation, technology transfer, management, and competitiveness [17, 18]. The most important foresight methods include Delphi technique, expert panel, literature review, scenario building and key/critical technologies [18].

Key technologies' studies are conducted in order to predict the future of technology through investigating experts’ opinions. This approach is usually applied to determine the priorities and key measures [19]. In this kind of studies, key technologies should have some specific features, such as policy-relevancy. In fact, a key technology or a related issue shows that the political decisions regarding that kind of technology may lead to desired outcomes. Discrimination is another characteristic of key technologies, *i.e*. key technologies should be clearly and transparently distinctive from other non-key technologies. In fact, the advancement of technology is not sufficient for selecting key technologies. Another feature of key technologies is reproducibility. The selection method for key technologies should be clear so that if someone who is not directly involved in identifying key technologies repeat the selection, similar results can be achieved [18-20].

The application of future studies in HIT was proposed in 2002 [21] and the necessity of using foresight to provide a basis for designing and constructing future health information systems was emphasized later in other publications [22]. According to the literature, conducting a foresight study has been suggested as an important tool to systematically investigate the future of HIT [21, 22]. In fact, by reviewing current trends and predictions, as well as employing tools for predicting and managing the future, health informatics professionals can be able to plan for the effective and innovative health information systems in the future [12]. Because the traditional methods of forecasting are not reliable approaches for future planning, systematic approaches, such as a future study and research methods can be used to assess the future to identify future trends and directions correctly [12]. Foresight studies are conducted to gain knowledge so that today’s decisions can be based more solidly on the available expertise. It is more than prognosis or prediction, and it holds the promise of managing uncertainty through intensive interaction between stakeholders and drawing a clear picture of the future [14].

Although conducting foresight studies can be helpful for future planning in the field of HIT, there has been very limited research in this area, especially in developing countries [23]. In 2012, Palvia *et al*. identified the key information technology issues in health care. They identified and prioritized ten key issues, such as the implementation of Electronic Medical Records, change management and quality assurance of electronic records by asking the opinions of two groups of managers and hospital Chief Executive Officers (CEOs) in the United States [24].

In 2014, Turan and Palvia identified the key HIT issues in Turkey. The results were formed based on the opinions of senior managers of the hospitals, and the key issues, such as the privacy, security and quality assurance of Electronic Health Records (EHR) were identified and prioritized [23]. In another study, Cresswell and Sheikh reported that five key HIT issues are the most significant areas over the coming decade. There issues were person- and patient-generated data, robotics and the automation of health care, innovative information infrastructures, smart facilities and the creation of learning health systems [8].

In Iran, few studies have been conducted to identify the key HIT issues for the future. One of them was initiated in 2007 and completed in 2010 as part of a comprehensive scientific health map for Iran. In this study, an HIT working group was formed and different methods, such as brainstorming were used to determine key HIT issues in the future. In this study, HIT perspectives, policies, and strategies were developed using trend analysis for a five-year time horizon. The four key HIT issues included immediate, cheap, stable, and secure access to the health records of the al society members, equitable access to health care resources and services, the use of knowledge management to improve the health and safety of the society members, and the development of governmental/central electronic services for the health system [25]. However, in this document, the key technologies were not discussed in detail.

In 2008, a pilot foresight study was conducted to identify the most appropriate technologies for Iran by 2025. The main aim of this project was to create a collection of scenarios about the most appropriate technologies for Iran and one of them was developing Electronic Health Records (EHR) for all members of the society [26]. However, in this study, the future technologies for the country were discussed in general, and only a small part of it was related to health services and its technologies [20]. Currently, Iran is moving towards implementing national electronic health records and a number of other HIT projects are completed across the country. However, it is not clear what the key technologies are, which one is more effective and what the potential obstacles are to achieve key technologies. Therefore, in this study, the researchers aimed to identify the key health information technologies and related issues for Iran until 2025.

## 2 METHODS

This qualitative study was part of the key technology approach [20] and was completed in 2016. The participants were selected by using purposive sampling method and included experts and the members of the HIT working group who helped with developing a comprehensive scientific health map for Iran in 2010 (N = 20). Having sent the invitation letters for conducting interviews, eventually, 13 people agreed to be interviewed by one of the researchers (MH) and data saturation was reached. In order to collect data, in-depth semi-structured interviews were conducted. Semi-structured interviews are in-depth interviews where the respondents have to answer preset open-ended questions [27]. In this study, prior to interviews, an interview guide was prepared based on the literature review. An interview guide is a schematic presentation of questions or topics and is used by the interviewer to conduct the interviews and comprise of the core questions [27]. The interview guide included questions about the current health information technologies in the country and the key health information technologies for the future. According to the national documents, four main areas of using these technologies were improving the accessibility of the individuals’ health records across the country, improving health equity, supporting and managing medical sciences, and improving e-health services. Moreover, the participants were asked to discuss the main areas that the technologies may influence and the main obstacles to achieve these technologies. The interviews were conducted in the interviewee's work place and lasted between 30 and 55 minutes.

The interviews were recorded using two MP3 recorders and if an interviewee did not permit recording his/her voice, notes were taken during the interview. The recording of the interview makes it easier for the researcher to focus on the interview content and the verbal prompts [27]. In the next stage, interviews were transcribed, and framework analysis was used to analyze the data. Framework analysis is a method that is used in the applied research in order to answer specific questions and includes five main steps: familiarization, identifying a thematic framework, indexing, charting, and mapping and interpretation [28, 29]. Finally, by comparing relationships, concepts, contradictions, and opinions the themes were extracted using MAXQDA 10.0 software.

To check the validity of results, ‘member checking’ was performed by sending a summary of findings to the participants to review and to verify themes. Additionally, the members of the research team rigorously reviewed the research process and the study details were documented carefully. Moreover, the participants had different background and work experiences in the field of HIT which supports the transferability of the results to a bigger sample size.

## 3 RESULTS

A total of 13 participants out of 20 took part in this study and the average time of the interviews was 44 minutes. The demographic characteristics of the participants including sex, age and work experience in the field of HIT are summarized in Table **1**. The main themes emerged from the findings are shown in Table **2**.

The first theme was immediate, cheap, stable, and secure access to the health records of the entire society. The key technologies related to this theme were National Health Information Network (NHIN), Electronic Health Records (EHR), national cloud-based service center, Personal Health Records (PHR), interoperability standards for electronic data exchange, and an infrastructure for information sharing across the public and private health care organizations. Regarding the first subtheme, most of the interviewees agreed that implementing NHIN with paying adequate attention to security and privacy issues was very important. For example, one interviewee stated:

“We still have not reached the goal of creating an NHIN. An extensive network that provides interoperability along with security and confidentiality and this issue can be considered a key issue. We need this technology to access information of all the society.”(M-2)

Implementing an integrated EHR to be accessible to the authorized organizations and the society members was found to be another key technology in the healthcare services. Although a lot of attempts have been made in this area, but the desired outcome has not achieved yet. In this context, an interviewee argued:

“Our main problem is still EHR. We may accept an online integrated electronic record as a new idea; however, the communication should be enhanced online. In any case, this technology should be integrated if it wants to be implemented properly.” (M-1)

Another key technology was a national cloud-based service center for integrating health data which may come from the public and the private sectors. In fact, the use of cloud-based services at a national center can be a solution for integrating heath data and seems to be achievable within the next ten years. In this regard, an interviewee stated:

“One of the major health field requirements is the development of a national data center that may need to be accessible by private sector. In fact, it should be such that all governmental and private centers can have access to it. We need integrity in the data center that requires proper planning” (M-3).

The development of Personal Health Records (PHR) with respect to security, confidentiality, and user-friendliness was found as another key technology by most interviewees. One of the interviewees believed that this technology is one of the key requirements to engage all the society members towards self-generated data. For this purpose, PHR should be designed “*user-friendly and secure*” (M-7). Another interviewee stated:

“We need a patient-centered file that makes it possible for the patient to access his/her information securely by automatically updated user-friendly software” (M-6).

Establishing interoperability standards for electronic data exchange across a variety of applications was another key technology. It seems that formulation and implementation of these standards are the main requirements for HIT planning within the next ten years. Concerning this issue, one of the interviewees indicated that:

“In the vast area of health sciences and in order to exchange information, some standards should be implemented,…., we should be thinking about interoperability between the applications to be installed on all devices and this will make the need for interoperability standards more critical” (M-7).

Another key technology issue was related to providing an infrastructure for information sharing across the public and private health care organizations. A number of interviewees stressed that exchanging data between different health information systems is crucial. According to one of the interviewees, this issue is of great importance in the developed countries and should be considered in the developing countries for the upcoming years (M-8).

Theme two was related to equitable access to health care resources and services. This theme included four key technologies as follows: telemonitoring technologies, large-scale remote healthcare services, and mhealth and its related technologies. Patients’ telemonitoring technologies, such as biometrics and sensors have been used widely in developed countries in recent years. One of the interviewees (M-2) alleged that in order to implement these technologies related infrastructure should be in place, but these technologies have not been implemented in our country and accurate planning is essential for simpler and more comprehensive collection of patients’ information. One participant stated:

“I think telemonitoring and the ability to produce data by patient is critical in the health care setting. This will not only improve the quality of care but also prevent the possible readmission” (M-13).

Regarding the development of remote healthcare services, one of the interviewees believed that it is a spectrum and may range from a simple electronic consultation between a doctor and a patient to a remote surgery (M-12). Another interviewee stated:

“Due to the multiple reasons, the original form of remote healthcare services has not been established in our country, but we should have proper planning to maximize our use of the potentials of this technology” (M-5).

One of the interviewees thought that there is a need to move towards mhealth technologies in the future and long-term planning is crucial in this context (M -5). Another interviewee stated:

“I can suggest mhealth development as an objective for the coming years in the health field that may entail even the details of mobile technology applications. Since, this technology is progressing rapidly, we will eventually draw into it, so it is wiser to have proper planning for the application of this technology” (M-10).

Theme three was about knowledge management in healthcare services through developing clinical decision support systems and creating social networks in the healthcare environment. Most of the interviewees believed that clinical decision support systems are key contributors in healthcare. Despite the global advances, in Iran, no significant progress has been made in this area mainly due to the lack of system integration. Advancement in the cloud-based services is another added feature for clinical decision support systems that makes accurate clinical decisions available quickly and comprehensively.

Regarding social networks in the healthcare environment, one of the interviewees (M-7) stressed that although several years have passed since the emergence of social networks, we have not yet exploited this technology. Another interviewee alleged: *“These social networks can be helpful for patients, their families and other citizens. We must use the capabilities of this technology in the upcoming years” (M-1).*

Theme four showed the importance of governmental/central electronic services for the health system. This theme consisted of three key technologies: an electronic health insurance system, business intelligence, and an integrated electronic monitoring system. Several interviewees considered the development of an electronic health insurance system as a key technology for Iran. Such a system can help to reduce costs, save time and improve efficiency and effectiveness. According to one of the interviewees:

“Making the insurance electroniclly and integrating it with EHR has been one of the aims in these years that unfortunately, has not been successful, so for the next ten years we must provide some measures to make it happen” (M-13).

Business intelligence was another key technology mentioned by the participants. This technology can be used in healthcare in order to collect and analyze clinical and financial data from different systems. One interviewee (M-1) believed that data collection and analysis can be improved through using business intelligence. He argued:

“Business intelligence has been used seriously in non-health sectors and has resulted in positive outcomes. Unfortunately, in the healthcare environment it has not been utilized properly. The information and data that can be gathered through these systems are very vital and potentially beneficial” (M-5).

Some of the interviewees also discussed the importance of having an integrated electronic monitoring system. Such a system can help to improve quality of care and performance across health centers. In this regard, one of the interviewees stated: *“health technologies seek integrated and systematic monitoring and evaluation which in turn requires a system to perform the intended evaluations, consistently”* (M-4).

Theme five was related to upgrading HIT infrastructure at the national level. This theme consisted of two key technologies: developing infrastructure for National Health Information Network (NHIN) and promoting basic infrastructure for mhealth. A number of interviewees emphasized that enhancing HIT infrastructure at the national level is crucial. Some of them noted that currently, there is a lack of a comprehensive infrastructure and this is a big challenge for the future of HIT in our country. In this regard, one of the interviewees maintained:

“Network infrastructure should be interoperable to be able to support collection, communication and analysis of data from multiple sources” (M-13).

According to the interviewees, mhealth and its related services and technologies are often expensive and it is one of the key issues for the future. One of the interviewees believed that cost-benefit analysis should be conducted and if it provides economic saving for us, necessary planning needs to be done in this area. Two participants explained their opinions as follows:

“The development of necessary infrastructure for promoting IT-based self-care programs is one of the key issues, and mhealth can provide self-care programs” (M-9).

“One of the most crucial discussions in the context of using mhealth is the development of infrastructure for this technology which has not yet been provided in our country” (M-10).

Theme six showed the areas that the mentioned technologies would influence and the main barriers to achieve these technologies. The interviewees agreed that the technologies could influence at the economic level (saving cost and increasing utilization), organizational level (facilitating communications between individuals and organizations), and individual level (improving quality of care and quality of life).

Theme seven showed that there were two main obstacles to achieve the technologies. Limited financial resources was the main economic obstacle and weaknesses in planning, technology acceptance by healthcare professionals and patients, stakeholders' involvement as well as cultural barriers in using health information technology were the main organizational obstacles.

## 4 DISCUSSION

The present study aimed to identify key health information technologies and related issues that Iran may face during the next decade. The first theme was related to instant, cheap, stable, and secure access to the heath records of the entire society. This could be achieved by implementing a number of technologies, such as NHIN, an integrated EHR, and PHR. Similarly, Valle *et al*. (2016) found that implementing NHIN was a key technology issue [30]. The implementation of an integrated EHR has also been addressed in other studies. For example, in a study conducted by Naderimanesh *et al*, it has been emphasized that all Iranians should have access to EHR by 2025 [26]. Other studies have similarly addressed the importance of implementation, security and confidentiality of EHR as a key HIT issue [23-25].

Another key technology was a national cloud-based service center for integrating health data and the importance of this key technology has been discussed in other studies [31, 32]. Concerning Personal Health Records (PHR), although this technology has not been considered in the previous foresight plans of Iran, in Turkey it has been regarded as one of the ten key technologies for the upcoming years [23]. Similarly, in other studies, the development of PHR has been considered as a key issue in order to improve quality of care [24, 33].

In order to provide equitable access to health care resources and services, the development of telemonitoring technologies, such as sensors and biometrics were found to be another key technology. These technologies are regarded as one of the most influential technologies in the next decade [8, 34]. According to the results, telehealth and mhealth services were found to be other key technologies for Iran until 2025. It is notable that these technologies have not previously been addressed or included in the scientific health map, health transform plan, and other policy documents of Iran. Similarly, in a study conducted by Mirakabad *et al*., mobile technology has been identified as a key technology for Iran; however, the importance of mhealth technology has not been addressed [35]. In contrast, Cresswell and Sheikh regarded mhealth as a key HIT issue, because it can help to collect data generated by patients and complete other technologies such as PHR [8].

The findings also showed that clinical decision support systems and social networks are other key technologies that should be taken into account. It is expected that these types of technology help to improve quality of care and patient safety. Although these technologies have been discussed in the comprehensive scientific health map of Iran, the findings of the present study revealed that the previous objectives have not been achieved yet. Similarly, in Turan and Palvia’s study, the use of decision support systems were found to be a key issue for hospitals, clinicians and patients in Turkey [23]. The importance of decision support systems was also addressed in Cresswell and Sheikh’s study; however, it was not regarded as one of the top five key technologies [8]. Governmental/central electronic services, such as an electronic health insurance system and an integrated electronic system were found to be other key technologies to monitor and evaluate healthcare services. The results are in line with the findings reported by Davari *et al*. who have discussed the importance of these technologies in their study [36]. Similarly, Deering emphasized the importance and the impact of these technologies on empowering patients and care givers [37].

According to the results, upgrading HIT and mhealth infrastructures were other key technologies which should be taken into account for the country. As these issues have not been considered in the previous domestic documents, it seems that it is time to pay more attention to them, as the technology is progressing and there will be more demands in the future. Similarly, in Cresswell and Sheikh’s study, implementing a new infrastructure has been highlighted as one of the five key technologies that will be the most important ones in the upcoming years [8]. In another study, Palvia *et al*. identified HIT infrastructure as one of the ten key issues for the future of United States. They also regarded telehealth infrastructure as a separate key issue [24].

Regarding the areas that the key technologies may influence, the results showed that technologies can influence the economic, organizational and individual levels. These results are in line with the findings reported in other studies [38-40]. Similarly, the findings related to the barriers to achieving key technologies (economic and organizational barriers) are supported with the results of other studies [41-43]. Overall, the results of the current study reflected the Iranian experts’ and stakeholders’ opinions about the key health information technologies and related issues of the country in the next decade. This was the first time in the country that such a foresight study was conducted in the field of HIT. Therefore, the results of this study can be considered as an important element for future technology strategy development processes. In addition, the results of the study can support public and private companies of Iran in terms of innovation, technology transfer, management, and competitiveness.

## 5 LIMITATIONS

This study was part of a foresight study which was conducted in the field of health information technology in Iran. As the key health information technologies can be different from one country to another and the results could be used by the policymakers, the results of the study seemed to be very important for the country and its future planning. However, this study had some limitations. In this study, the method of purposive sampling was used to invite the experts and the members of the HIT working group. However, a limited number of participants agreed to be interviewed. As these participants were the key informants and their perspectives and experiences were of great value to identify key health information technologies for the country, the authors believe that the results derived from this study were rich enough to be used in the future research or in practice. Moreover, in a qualitative study, generalizability of the results is not the main goal, but rather to provide a rich, contextualized understanding of the human experience through the intensive study of particular cases.

Although key health information technologies and related issues were identified in this study, it was not clear which technology was the most important one for the future. Therefore, in order to identify the level of importance, different research methods, such as Delphi technique should be used to reach consensus for prioritization and for better resource allocation.

## CONCLUSION

This study aimed to identify the key health information technologies and related issues for Iran until 2025. The findings revealed that the development of NHIN, EHR, PHR, a cloud-based service center, interoperability standards, patient monitoring technologies, telehealth, mhealth, clinical decision support systems, HIT and mhealth infrastructure were found to be the key technologies for the future. The results of the current study can be used to develop a roadmap to guide the development of health information technology in the future. In the future research, foresight studies can be conducted by using different approaches, such as scenario building and trend analysis. Moreover, foresight studies can be conducted for each technology to obtain a more detailed picture of the future.

## Figures and Tables

**Table 1:** Characteristics of the participants.

Variables		Frequency (%)
Sex	Men	12 (92.3%)
	Women	1 (7.7%)
Age	30-39	1 (7.7%)
	40-49	5 (38.5%)
	50-59	7 (53.8%)
Work experience in HIT	1-5	4 (30.8%)
	6-10	6 (46.2%)
	11-15	2 (15.4%)
	16-20	1 (8%)

**Table 2:** Themes and sub-themes related to key health information technologies and issues.

**Themes**	**Sub-themes**
**Immediate, cheap, stable, and secure access to the health records of the entire society**	National Health Information Network
	Electronic Health Records
	National cloud-based service center
	Personal Health Records
	Interoperability standards for electronic data exchange
	Infrastructure for information sharing
**Equitable access to health services and resources**	Telemonitoring technologies
	Large-scale remote health services
	mhealth and its related technologies
**Knowledge management in healthcare services to improve quality of care and patient safety**	Clinical decision support systems
	Social networks in healthcare environment
**Governmental/central electronic services for the health system**	Electronic health insurance system
	Business intelligence
	Integrated electronic monitoring system
**Upgrading HIT infrastructure at the national level**	Infrastructure for NHIN
	Infrastructure for mhealth
**Areas that technologies may influence**	Economical level
	Organizational level
	Individual level
**Obstacles to achieve technologies**	Economic obstacles
	Organizational obstacles
